# Assessing signs and impacts of achondroplasia: psychometric evaluation of the Achondroplasia Child Experience Measures

**DOI:** 10.1186/s41687-026-01123-z

**Published:** 2026-06-19

**Authors:** Meryl Brod, Lori McLeod, Alden Smith

**Affiliations:** 1 The Brod Group, Mill Valley, USA; 2 RTI Health Solutions, Research Triangle Park, USA; 3 Ascendis Pharma, LLC, Palo Alto, USA

**Keywords:** Achondroplasia, Surveys and questionnaires, Psychometrics, Caregivers, Child

## Abstract

**Background:**

Achondroplasia, although rare, is the most common genetic form of skeletal dysplasia and is associated with a wide range of clinical manifestations and complications throughout the body. This study evaluated the psychometric measurement properties of the Achondroplasia Child Experience Measures (ACEMs), which were previously conceptually developed as condition-specific, observer-reported outcome measures to assess the signs (ACEM–OSM) and impacts (ACEM–Impact) of achondroplasia in children aged 2 to < 12 years.

**Methodology:**

Data for the psychometric evaluation were from a non-interventional, observational study (*N* = 200 (impacts) and *N* = 148 (signs)) and a phase 2, multicenter, double-blind, randomized, placebo-controlled, dose escalation interventional trial (*N* = 57) of navepegritide (TransCon CNP) administered once weekly. The larger observational study sample size allowed for evaluation of distributional properties, test-retest reliability, and construct validity. Trial data was analyzed to confirm reliability and validity findings and evaluate responsiveness.

**Results:**

Factor analysis and qualitative results identified six domains for the ACEM–Impact and one domain for the ACEM–OSM. The psychometric properties of both measures were found to be acceptable for reliability and validity for the intended use. An 8-item ACEM–OSM and a 25-item ACEM–Impact were finalized.

**Conclusions:**

The ACEMs are reliable and valid measures of the signs and impacts of achondroplasia on children. Incorporation of these measures in clinical and research settings can aid in assessing new treatments and improve our understanding of the experience of those living with achondroplasia.

**Trial registration:**

National Institutes of Health Clinicaltrials.gov (ID NCT04085523) and European Union Drug Regulating Authorities Clinical Trials database (EudraCT 2019-002754-22).

**Supplementary Information:**

The online version contains supplementary material available at https://doi.org/10.1186/s41687-026-01123-z.

## Background

Achondroplasia is a rare condition; however, it is the most common genetic form of skeletal dysplasia and affects more than 250,000 people globally with prevalence of 4.7 per 100,000 population [1–6]. The incidence of achondroplasia is estimated to be between one in 10,000 and one in 30,000 live births [6]. Achondroplasia has historically been considered a bone growth disorder. However, achondroplasia is associated with a wide range of clinical features and complications in addition to the well-characterized skeletal dysplasia [6, 7]. Clinical features of achondroplasia typically include small stature, disproportionality such as shortening of the legs and arms, macrocephaly, small chest size, curvature of the spine (kyphosis, lordosis), short fingers and trident-shaped hands, joint hypermobility in hips and knees, low muscle tone with weakness [8], and bowing of the legs [9]. Frequently observed co-morbidities and complications include obstructive sleep apnea, middle ear dysfunction, spinal stenosis, obesity, impaired hearing, respiratory issues, reduced muscle strength and stamina, and increased mortality rate [6, 9–12].

The clinical features and complications of achondroplasia in children have been well-studied [9, 11]. The impacts of these features and complications are less well studied and can impact a child’s well-being, daily functioning, emotional health, and social health [13–18].

Unfortunately, there are limited valid and reliable condition-specific measures available to assess the impacts of these clinical complications on the functioning, emotional well-being, and social well-being of children with achondroplasia [13, 17–21]. The few measures that do exist and that have been used in children with achondroplasia are, unfortunately, not optimal condition-specific measures due to methodological and conceptual concerns.

To address these gaps, the Achondroplasia Child Experience Measure-Observable Signs Measure (ACEM–OSM) (formerly named the ACEM–Symptom) and the Achondroplasia Child Experience Measure–Impact (ACEM–Impact) were generated. Measure items were based on concept elicitation interviews with parents of children aged 2 < 12 years (*N* = 36), children aged 9 to < 12 years (*N* = 13) with achondroplasia, 7 clinical expert interviews and tested for relevance and comprehension in cognitive debriefing interviews (*N* = 16) [13] according to the United States (US) Food and Drug Administration (FDA) guidelines for the development of patient-reported outcome (PRO) measures [22]. The original framework of the ACEM–Impact consisted of the domains of functioning, emotional well-being, social well-being, and need for assistance/adaptive devices while the ACEM-OSM consisted of 1 domain of symptoms. Both measures are observer-reported outcome (ObsRO) measures in which the caregiver living with the child with achondroplasia completes the measures. Further, as caregivers may not all be similarly trained on how to identify the signs of achondroplasia in their children, a guide for clinicians to review with the parents was also developed along with the ACEM–OSM. Caregivers living with a child with achondroplasia were chosen to complete the measures as an ObsRO, rather than the children themselves as a patient-reported outcome measure. While there is evidence of increased reliability and validity of health status reports among children aged 8 through 11 years compared to younger children, there is considerable variation in the cognitive abilities, understanding, and attention, which are required to complete a health-related PRO instrument, among children of this age [23]. An analysis of the concept elicitation interview/focus group transcripts of the child participants aged 9 to < 12 years suggested that some of these children had difficulties articulating their thoughts and/or had trouble focusing their attention on the interview questions. Further, as many of the common complications and impacts may not occur in the presence of health care providers, caregivers are likely the best reporters of these issues. Based on this evidence, the decision was made to develop ObsRO measures, rather than child PROs.

The purpose of this study was to evaluate the psychometric measurement properties of the ACEM–OSM and the ACEM–Impact measures, which included examination of item-level descriptive statistics, structure, reliability, validity, and responsiveness of the measures.

## Methods

### Overview

Data from a non-interventional, observational, web-based validation study [24, 25] (caregivers of children with achondroplasia aged 2 to < 12 years), conducted in the US, the United Kingdom, Australia and Spain, provided the primary data set for the psychometric evaluation of the 8-item ACEM–OSM and 31-item ACEM–Impact measures. The observational study was launched in March 2020, with recruitment halted during the COVID-19 pandemic and recruitment ending in June 2022. At the time of the observational study, there was no approved pharmacological treatment for achondroplasia available. The psychometric results were then confirmed using data (caregivers of children with achondroplasia aged 2 to < 11 years) from a phase 2, multicenter, double-blind, randomized, placebo-controlled, dose escalation interventional trial evaluating the safety, efficacy, and pharmacokinetics of navepegritide administered once weekly for 12 months in prepubertal children with achondroplasia (ACcomplisH Trial) [26]. The interventional trial was launched in June 2020 and data for the psychometric analyses was collected in 8 countries (US, Ireland, Germany, Denmark, Austria, Portugal, Australia and New Zealand). The non-interventional, observational study, with its larger sample size, allowed for a robust evaluation of the distributional properties, scoring algorithm, test-retest reliability, and construct validity. Analysis of the interventional trial data provided an opportunity to confirm the reliability and validity findings from the non-interventional, observational study and to extend the evaluation to include the longitudinal property of responsiveness. Sample size for the interventional study was based on the primary purpose of the study which was efficacy rather than psychometric validation. The sample size for the observational study was selected based on a balance between obtaining an adequate sample for test-retest and recruitment demands.

In order to conduct the psychometric analyses, a validation battery was developed. The battery was administered electronically in both studies (see Supplementary file 1, battery of measures). In the non-interventional, observational, web-based study, the complete battery was administered at a single timepoint. All survey participants were also invited to complete an online retest administration of the ACEM–OSM and ACEM–Impact along with a single question regarding changes in major life events. In the interventional trial, the battery was administered at baseline and week 52 (visit 7) or at early termination.

For the ACEM–OSM data set, the observational study commenced with an original version of ACEM–OSM items (referred to as the original ACEM–OSM). However, after the study was launched, FDA feedback was received requesting that examples for each sign in the measure be included. No changes in the actual signs assessed in the measure were made. Wording for the items was revised to include examples, and the observational study was re-launched with the adapted ACEM–OSM items. For example, the pre-adapted version for the item “Balance issues” had no examples. In the adapted version, “Balance issues” was followed by “(such as falls/trips often and/or thrown off balance easily)”. The validation data set presented here represents the findings from the observational study sample who completed the adapted version (*N* = 148). The interventional trial sample all completed the original version of the measure. In the interventional trial, all measures in the battery were completed by a caregiver before any other clinical assessments and the same caregiver was expected to complete the measures throughout participation in the interventional trial. For the ACEM–Impact, no changes were made in the measure after the observational study launch and so the data set included all respondents to the survey (*N* = 200, inclusive of the 148 respondents who completed the adapted version of the ACEM–OSM).

In the non-interventional, observational, web-based survey study, key inclusion criteria were:

- Have a child diagnosed with achondroplasia between the ages of 2 and < 12 years.
- Be an adult, aged 18 years or older.
- Be able to read, write, and speak English or Spanish (Spain).
- Be a parent or guardian of the child and actively involved in the child’s day-to-day care.

In the ACcomplisH, interventional trial, key inclusion criteria were:

- Having a child with a clinical diagnosis of achondroplasia with genetic confirmation.
- Child with achondroplasia aged between 2 to < 11 years at screening visit.
- Child of prepubertal stage (tanner stage 1 breasts for girls or testicular volume < 4 mL for boys) at screening visit.
- Child able to stand without assistance.
- Caregiver willing and able to administer subcutaneous injections of study drug.

### Analytic methods

The analyses were conducted using SAS [27]. Each ACEM score was computed as the average of the non-missing item-level score when at least 1/2 of the item scores are non-missing. Otherwise, the score was not computed. Either a raw score or a transformed score for the ACEM–Impact can be computed as a linearly transformed 0-to-100 score by dividing the raw score by the maximum response category for the domain (either 4 or 5) and multiplying by 100. Either a raw ACEM–OSM total score or a transformed score can be computed as a linearly transformed 0-to-100 score by dividing the raw score by 4 and multiplying by 100. The ACEM–OSM total score is scored as one domain and the ACEM–Impact total score is computed as the average of the six transformed domain scores. For supporting measures missing data was scored based on the developer’s rules for that measure. No imputation was used for any of the analyses.

Item-level descriptive statistics and response frequency distributions were tabulated. The percentage of participants with the lowest and highest possible item scores were evaluated for potential floor and ceiling effects—defined as twice the expected probability for a uniform distribution (e.g., 40% responding in an extreme response category for a 5-point ordinal response scale).

As an examination of the relationships among the ACEM–OSM items and ACEM–Impact items, inter-item polychoric correlations among the items were computed. Pairs of items with high inter-item correlation (|*r*| > 0.80) were flagged for possible redundancy and pairs of items with low inter-item correlation (|*r*| < 0.30) were flagged as potentially not sufficiently related enough to warrant inclusion in a summary score. Item-total correlations (corrected to remove the specific item score) were computed for every item; correlation coefficients of at least 0.40 were considered adequate. The sample size for each inter-item correlation was based on the pairs that were non-missing.

#### Factor structure

Both the comparative fit index [28] and non-normed fit index [29] were used as criteria for confirmatory factor analysis (CFA) model fit to evaluate the hypothesized domain structures of the ACEM–OSM and ACEM–Impact. For both of these fit indices, values greater than 0.95 were most desirable for controlling type I and type II error rates; values between 0.90 and 0.95 were considered marginal; and values lower than 0.90 indicated poor model fit [30]. In addition, the root mean square error of approximation (RMSEA), another useful indicator of model fit was examined. For this index, values smaller than 0.06 suggest satisfactory model fit values, values of 0.06 but less than 0.08 indicate fair model fit, values of 0.08 to 0.10 are mediocre, and values greater than 0.10 suggest poor fit [30–32]. Also, residual variances should be nonnegative, indicating that the imposed number of factors are not redundant for the data.

#### Internal consistency

Internal consistencies were computed using Cronbach’s [33] coefficient alphas. The approximate range of optimal alphas suggested by Streiner and Norman [34] is between 0.70 and 0.90. Cronbach’s alpha was computed on complete item-level data.

#### Convergent and divergent validity

The magnitude and direction of the resulting Pearson correlation coefficients were compared with respect to specific a priori hypotheses and to Cohen’s [35] guideline for interpreting correlation coefficients: absolute values of correlations of 0.50 or greater were considered strong, correlations that fall between 0.30 and 0.49 were moderate, and those that fall between 0.10 and 0.29 were considered small. It was anticipated that the ACEM–OSM and ACEM–Impact scores and supporting PRO measures describing similar concepts would be more strongly associated (convergent validity) and therefore, exhibit stronger correlation coefficients than items describing dissimilar signs (divergent validity). The Patient-Reported Outcomes Measurement Information System (PROMIS) [36, 37] was used to assess validity for the ACEM–OSM as it assesses global health, which is conceptually related to symptoms and the Quality of Life in Short Stature Youth (QoLISSY) [38] was used as it assesses impacts of short stature and thus, is conceptually similar to the ACEM–Impact. The Patient Global Impression of Severity (PGIS)(proxy report) was used for both measures as it included assessment of both symptom and impact concepts.

#### Known-groups validity

Analyses of variance (ANOVA) examined mean differences in the ACEM–OSM and ACEM–Impact Total score across subgroups, defined by each measure’s rating scale, using data at baseline in the observational study and screening and visit 7 in the trial data. Measures were chosen which assessed the concepts in the hypothesized relationships for testing known-groups validity.

The known-groups validity of the ACEM–OSM was examined using the 10-item Short Form Health Survey (SF-10) for Children (proxy), acute version [39] which assesses the perceived health status (child’s health and well-being)(rating scales: 4- and 5-point Likert scales with 5 variations on response options); the SF-10 Global Health item, which is a global rating of symptom severity; the Pediatric Quality of Life Inventory (PedsQL) 4.0 Generic Core Scales – Parent version [40] scores which assesses QoL of child (rating scale: 5-point Likert scale from 0 (never) to 4 (almost always)); and the PGIS-Child Symptom, a 1-item rating which assesses symptom severity. For the ACEM–Impact, the SF-10 Physical Summary Score, which assesses physical health and the PedsQL Physical domain, which assesses physical functioning; the Patient-Reported Outcomes Measurement Information System (PROMIS) Parent Proxy Upper Extremity which assesses child’s ability to conduct activities with their upper extremities (rating scale: 5-point Likert scale ranging from 5 (with no trouble) to 1 (not able to do)) and the Satisfaction with Life Scale – Child (proxy) measures, assessing child’s satisfaction with life (rating scale: 5-point Likert scale from 1 (disagree a lot) to 5 (agree a lot)) were used.

The hypothesized relationships between which subgroups were relevant for each analysis and the definitions for the subgroups are shown in tables S2 and S6 in Supplementary file 2.

#### Test-retest reliability

Intraclass correlation coefficients (ICCs) were computed using data collected at two consecutive timepoints (observational study: baseline to 2-week retest; interventional trial: screening visit (1–4 wks. prior to randomization) to baseline visit (wk. 0) and again at visit 3 (wk. 8) to visit 4 (wk. 12)) for the following groups of patients: (1) overall sample, (2) those with major life events based on their response to the question “Have you and/or your child experienced any major life events in the *past two weeks* (such as moving, divorce, school or job change, birth or adoption of another child)?”, and (3) those with no change in corresponding PGIS item responses.

Intraclass correlation coefficients were computed using a 2-way (subjects × time) mixed-effects ANOVA with absolute agreement for single measures [41]. It is generally recommended that ICCs be at least 0.70 for multi-item scales [42]. In addition to the ICCs, scatterplots were used to depict the agreement of the scores at the two timepoints.

#### Ability to detect change (Interventional trial data)

Responsiveness was evaluated using change scores computed with data from the interventional trial from screening and visit 7. Change scores were computed and compared across groups formed using corresponding PGIS ratings. Specifically, descriptive statistics paired t-tests and *P* values computed. The a priori hypothesis tested was that those who reported improvement on the corresponding PGIS item would show greater improvement in ACEM–OSM and ACEM–Impact compared with participants who reported no change or worsening.

## Results

### Child characteristics

Table 1 presents sample characteristics of the children of the caregivers at baseline for the observational study and at screening for the interventional trial.

**Table 1:** Child characteristics

Patient characteristic	Observational study		Interventional trial
	ACEM–Impact Data Set^†^	ACEM–OSM Data Set^‡^	
**Age**			
n	200	148	57
Mean (SD)	6.66 (2.7)	6.98 (2.6)	5.42 (2.7)
Median, Minimum–Maximum	7.0, 2.0–11.0	7.0, 2.0–11.0	5.0, 2.0–10.0
**Sex**,** n (%)**			
n	200	148	57
Female	89 (44.5)	68 (45.9)	33 (57.9)
Male	111 (55.5)	80 (54.1)	24 (42.1)
**What is your child’s height? (cm)**			
n	192*	141*	42*
Mean (SD)	101.52 (23.9)	105.66 (25.9)	86.81 (12.9)
Median, Min-Max	96.5, 62.0-200.0	101.6, 62.0-200.0	84.7, 66.0-107.8
**Race and ethnicity**^**§**^, **n (%)**			
Asian	10 (5.0)	7 (4.7)	6 (10.5)
Black/African American	25 (12.5)	25 (16.9)	2 (3.5)
Hispanic/Chicano/Latino	13 (9.2)	4 (3.6)	6 (10.5)
Native American/Native Alaskan	0 (0.0)	0 (0.0)	—
Native Hawaiian/Other Pacific Islander	2 (1.4)	1 (0.8)	1 (1.8)
White/Caucasian	153 (76.5)	115 (77.7)	48 (84.2)
Mixed or multiple	2 (6.7)	2 (6.7)	—
Other race or ethnicity not listed	1 (0.5)	0 (0.0)	—
Ethnicity unknown/not reported	—	—	2 (3.5)
Prefer not to answer	4 (2.0)	2 (1.4)	—
**When was your child diagnosed with ACH?, n (%)**			
n	200	148	42
In utero (before birth)	72 (36.0)	38 (25.7)	16 (38.1)
At birth	41 (20.5)	37 (25.0)	9 (21.4)
After birth	85 (42.5)	73 (49.3)	17 (40.5)
Don’t know	2 (1.0)	0 (0.0)	0 (0.0)
**If diagnosed after birth**,** what age (weeks) was your child when were diagnosed?**			
n	85	73	17
Mean (SD)	9.93 (13.1)	7.58 (8.5)	12.06 (12.2)
Median, Minimum–Maximum	5.0, 1.0–77.0	5.0, 1.0–46.0	8.0, 0.0–40.0
**Have you or your child experienced any major life events in the past 2 weeks?** ^**¶**^, **n (%)**			
n	177*	132*	47*
Yes	47 (26.6)	39 (29.5)	2 (4.3)

ACEM–Impact = Achondroplasia Child Experience Measure–Impact; ACEM–OSM = Achondroplasia Child Experience Measure–Observable Signs Measure; ACH = achondroplasia; SD = standard deviation

^†^ No changes were made in the ACEM–Impact after the observational study launch, so sample includes all respondents

^‡^ In response to FDA comments, changes were made in the ACEM–OSM after the observational study launch, so sample includes those who completed the adapted ACEM–OSM

^§^ Respondent could report more than one category if applicable. Additionally, the categories varied by country

^¶^ The question regarding major life events was answered at retest in the observational study

*Smaller n due to missing data

Note: Percentages of categorical variables are calculated using all responses

### ACEM–OSM results

#### Item-level descriptive statistics

For the observational study data, descriptive statistics of the ACEM–OSM showed no evidence of problematic floor (worst status) or ceiling (best status) effects for any of the ACEM–OSM items, with no more than 35.4% of participants responding at the extremes of the score ranges, thus allowing scores to demonstrate either improvement or worsening from baseline.

For the interventional trial data, each of the eight items demonstrated ceiling (best state) effects with more than 40% selecting the best state response category at screening.

#### Inter-item correlations

All ACEM–OSM inter-item correlations are acceptable, within the proposed range of 0.30 to 0.80. All item-total correlations are greater than 0.40 and satisfactory, ranging from 0.57 to a maximum of 0.79 (Supplementary file 2, Table S1).

#### Confirmatory factor analysis results

A 1-factor CFA model was fit to the 8-item ACEM–OSM. Table 2 provides the estimated standardized factor loadings and key fit statistics. The comparative fit index (CFI) (0.99) and Tucker-Lewis index (TLI) (0.99) were both greater than 0.95, suggesting satisfactory model fit. The RMSEA (0.07) was between 0.06 and 0.08, suggesting fair model fit. Further, all standardized factor loadings were greater than or equal to 0.65, indicating that all items were adequately related to the latent symptoms factor. Together, these results provide strong support for the appropriateness of an ACEM–OSM total score.

**Table 2:** One-factor CFA model for ACEM–OSM

ACEM–OSM item	Standardized estimates (standard errors)
**Factor 1**	
Ear problems	0.706* (0.049)
Pain	0.650* (0.051)
Sleep apnea	0.701* (0.043)
Trouble breathing	0.873* (0.029)
Hearing problems	0.851* (0.035)
Low stamina	0.655* (0.049)
Balance issues	0.666* (0.053)
Speech issues	0.711* (0.046)
**Fit statistics**	
Chi-square, *df*, *P*	34.347, 20, 0.024
RMSEA	0.070
CFI	0.989
TLI	0.985
SRMR	0.032

ACEM–OSM = Achondroplasia Child Experience Measure-Observable Signs Measure; CFA = confirmatory factor analysis; CFI = comparative fit index; *df* = degrees of freedom; RMSEA = root mean square error of approximation; SRMR = standardized root mean residual; TLI = Tucker-Lewis index

#### Scale-level internal consistency reliabilities

The Cronbach’s alphas were highly satisfactory: 0.88 (*n* = 141, observational study).

#### Convergent/divergent validity

It was hypothesized that the ACEM–OSM score would correlate moderately to strongly with PGIS-Child Symptom ratings and with the PROMIS-Parent Proxy [36, 37] Global Health total scores. In fact, the correlations, in the observational study data, with the PROMIS Global Health total scores were moderate (*r* = − 0.45) and the correlations with the PGIS-Child Symptom ratings were strong (*r* = 0.62). It is noteworthy that the correlations between the ACEM–OSM score and the PGIS-Child Impact ratings were smaller in magnitude—this supplies evidence of divergent validity for the ACEM–OSM score. In the trial data, the ACEM–OSM score correlations with the PGIS-Child Symptom ratings were strong (screening = 0.70, visit 7 = 0.66) and correlations with the PROMIS Global Health total scores were moderate to strong (screening = − 0.48, visit 7 = − 0.53). Thus, overall, there is adequate convergent and divergent validity evidence for the ACEM–OSM scores.

#### Known-groups validity

In the observational study data, using the SF-10 Global Health item, the overall test was statistically significant (*P* < 0.001) and all item response categories (with *n* ≥ 5) followed the expected pattern. Caregivers who reported an “excellent” or “good” rating on the SF-10 Global Health item had lower mean ACEM–OSM scores compared with caregivers who provided a “fair” rating on the SF-10 Global Health item. Additionally, caregivers who provided an “excellent” rating on the SF-10 Global Health item had lower mean ACEM–OSM scores than caregivers who reported “very good” on the SF-10 Global Health item (*P* = 0.03).

Using the PGIS-Child Symptom rating, the overall test was also statistically significant (*P* < 0.001), and all response categories followed the hypothesized pattern such that caregivers who reported better (lower) PGIS-Child Symptom ratings had lower mean ACEM–OSM scores than caregivers who reported worse (higher) PGIS-Child Symptom ratings.

In the interventional trial data, using the PGIS-Child Symptom rating, the overall test was statistically significant (*P* < 0.001), and all participant groupings (with *n* ≥ 5) followed the hypothesized pattern such that caregivers who reported better (lower) PGIS-Child Symptom ratings had lower mean ACEM–OSM scores than caregivers who reported worse (higher) PGIS-Child Symptom ratings; however, no pairwise comparisons were statistically significant at visit 7 (likely influenced by small sample sizes in some groups).

Overall, the results provide support for known groups validity for the ACEM–OSM score (Supplementary file 2, Table S2).

### Test-retest reliability

ICCs for the scores all exceeded the recommended minimum of 0.70 for multi-item scales [42], indicating satisfactory levels of reliability (Supplementary file 2, Table S3).

### Ability to detect change

As shown in Table 3, using the PGIS items as external criteria, the mean differences between PGIS improved group versus the PGIS no change/worsening group were statistically significant for ACEM–OSM (by PGIS-Child Symptom).

**Table 3:** ACEM–OSM ability to detect change

Transformed score	Improved group			No change/worsening group			t-statistic (*P* value)
	*n*		LS mean (SE), median, min, max, (95% CI)	*n*		LS mean (SE), median, min, max, (95% CI)	
ACEM–OSM Total by PGIS-Child Symptom	11	−6.86 (3.3), − 9.4, − 21.9, 15.6, (− 14.79–1.07)		26	6.86 (2.2), 3.1, − 6.3, 35.4, (2.53–11.18)		−3.46 (0.002)

ACEM–OSM = Achondroplasia Child Experience Measure-Observable Signs Measure; CI = confidence interval; LS = least squares; PGIS = patient global impression of severity; SE = standard error

### ACEM–Impact results

#### Item-level descriptive statistics

For the observational study data, descriptive statistics of the 31 ACEM–Impact items showed no evidence of problematic floor (worst status) or ceiling (best status) effects for any of the ACEM–Impact items, with no more than 33.3% of participants responding at the extremes of the score ranges, thus allowing scores to demonstrate either improvement or worsening from baseline.

For the interventional trial data, 14 items demonstrated ceiling (best state) effects with more than 40% selecting the best response category at screening.

#### Inter-item correlations

The inter-item polychoric correlations and item-total correlations among the ACEM–Impact items are shown in Table 4 and are organized based on the initial conceptual framework from the development phase. The inter-item correlations among the ACEM–Impact Daily Living Functioning items ranged from a minimum of 0.36 to a maximum of 0.78. For the ACEM–Impact Physical Functioning items, the minimum inter-item correlation was 0.57; the maximum correlation was 0.75. Importantly, the inter-item correlations between the ACEM–Impact Daily Living Functioning items and the ACEM–Impact Physical Functioning items were lower. These correlations ranged from 0.12 to 0.19 to a maximum of 0.58.

The ACEM–Impact Emotional Well-being items were quite strongly intercorrelated. The minimum correlation was 0.64. However, there were 3 inter-item correlations of 0.80 or above: 0.83 between Item 2e (item 5: Worried) and Item 2d (item 4: Sad), 0.81 between Item 2f (item 6: Embarrassed) and Item 2e (item 5: Worried), and 0.80 between Item 2f (item 6: Embarrassed) and Item 2d (item 4: Sad).

The 3 ACEM–Impact Social Functioning items were strongly intercorrelated, with a minimum correlation of 0.62 and a maximum correlation of 0.73. Similarly, the 3 ACEM–Impact Social Well-being items were strongly intercorrelated, the minimum correlation being 0.64 and the maximum intercorrelation being 0.76. It is noteworthy that the inter-item correlations between the ACEM–Impact Social Functioning items and the Social Well-being items were lower, ranging from 0.34 to 0.56. The minimum inter-item correlation between the 3 ACEM–Impact School items was 0.46; the maximum was 0.67.

Overall, the item-total correlation values supported the results based on the inter-item correlations, suggesting modifications of the proposed conceptual framework for the ACEM–Impact items.

**Table 4:** ACEM–Impact inter-item correlations, baseline (*n* = 100 to 199)

	Daily Living Functioning					Physical Functioning					Emotional Well-being						Social Functioning			Social Well-being				School		
	DLF1	DLF2	DLF3	DLF4	DLF5	PF1	PF2	PF3	PF4	PF5	EWB1	EWB2	EWB3	EWB4	EWB5	EWB6	SF1	SF2	SF3		SWB1	SWB2	SWB3	S1	S2	S3
DLF1	—																									
DLF2	0.61*	—																								
DLF3	0.49*	0.78*	—																							
DLF4	0.36*	0.44*	0.57*	—																						
DLF5	0.37*	0.66*	0.75*	0.60*	—																					
PF1	0.41*	0.41*	0.45*	0.51*	0.58*	—																				
PF2	0.26*	0.36*	0.47*	0.47*	0.57*	0.61*	—																			
PF3	0.19*	**0.12**	0.27*	0.52*	0.49*	0.65*	0.69*	—																		
PF4	0.46*	0.51*	0.47*	0.43*	0.51*	0.58*	0.64*	0.57*	—																	
PF5	0.25*	0.31*	0.43*	0.56*	0.57*	0.61*	0.71*	0.74*	0.75*	—																
EWB1	**0.12**	**-0.03**	**0.04**	0.33*	0.17*	0.28*	0.37*	0.49*	0.27*	0.38*	—															
EWB2	0.17*	**0.08**	**0.14**	0.37*	0.23*	0.41*	0.36*	0.54*	0.33*	0.39*	0.70*	—														
EWB3	**0.08**	**0.01**	**0.13**	0.33*	0.28*	0.42*	0.38*	0.52*	0.26*	0.41*	0.70*	0.78*	—													
EWB4	**0.04**	**-0.14**	**0.00**	0.38*	**0.14**	0.32*	0.36*	0.54*	0.24*	0.40*	0.73*	0.78*	0.77*	—												
EWB5	**0.07**	**-0.08**	**0.02**	0.40*	0.22*	0.36*	0.43*	0.62*	0.32*	0.49*	0.73*	0.76*	0.77*	0.83*	—											
EWB6	**-0.05**	-0.22*	**-0.07**	0.33*	**0.09**	0.37*	0.39*	0.56*	**0.12**	0.42*	0.64*	0.69*	0.77*	0.80*	0.81*	—										
SF1	0.55*	0.37*	0.44*	0.40*	0.43*	0.50*	0.59*	0.52*	0.54*	0.50*	0.48*	0.40*	0.38*	0.27*	0.34*	0.24*	—									
SF2	0.22*	0.19*	0.40*	0.44*	0.38*	0.49*	0.50*	0.56*	0.40*	0.56*	0.55*	0.57*	0.57*	0.52*	0.56*	0.54*	0.62*	—								
SF3	0.31*	0.25*	0.34*	0.45*	0.38*	0.39*	0.50*	0.53*	0.44*	0.51*	0.53*	0.46*	0.40*	0.39*	0.43*	0.35*	0.73*	0.69*	—							
SWB1	**-0.07**	**-0.14**	**0.04**	0.36*	0.18*	0.43*	0.48*	0.67*	0.27*	0.53*	0.68*	0.57*	0.64*	0.65*	0.72*	0.71*	0.36*	0.56*	0.35*		—					
SWB2	**0.08**	**-0.06**	**0.06**	0.26*	0.17*	0.30*	0.30*	0.48*	0.27*	0.41*	0.65*	0.60*	0.49*	0.54*	0.55*	0.54*	0.34*	0.54*	0.39*		0.64*	—				
SWB3	**0.12**	**-0.07**	**0.13**	0.42*	0.23*	0.40*	0.39*	0.56*	0.27*	0.44*	0.56*	0.58*	0.56*	0.60*	0.66*	0.62*	0.40*	0.54*	0.43*		0.76*	0.70*	—			
S1	**0.04**	0.21*	0.41*	0.54*	0.54*	0.40*	0.65*	0.62*	0.36*	0.58*	0.54*	0.68*	0.65*	0.68*	0.69*	0.68*	0.44*	0.65*	0.66*		0.61*	0.48*	0.58*	—		
S2	0.32*	0.32*	0.52*	0.57*	0.52*	0.63*	0.55*	0.52*	0.48*	0.59*	0.40*	0.48*	0.48*	0.43*	0.50*	0.48*	0.56*	0.72*	0.64*		0.33*	0.42*	0.47*	0.63*	—	
S3	**-0.01**	**0.10**	**0.18**	0.43*	0.38*	0.40*	0.53*	0.52*	0.25*	0.43*	0.37*	0.50*	0.54*	0.59*	0.60*	0.60*	0.29*	0.53*	0.40*		0.53*	0.32*	0.50*	0.67*	0.46*	—
I-t	0.51	0.75	0.79	0.54	0.68	0.66	0.72	0.72	0.68	0.78	0.74	0.78	0.81	0.83	0.84	0.79	0.69	0.65	0.73		0.71	0.66	0.77	0.71	0.52	0.57

* *P* < 0.05

ACEM–Impact = Achondroplasia Child Experience Measure-Impact; DLF = Daily Living Functioning; I-t = item-total correlations; PF = Physical Functioning; EWB = Emotional Well-being; SF = Social Functioning; SWB = Social Well-being; S = School

Note: **Bold text** indicates *r* < 0.30. Item-total correlations are displayed in the last row of the matrix

#### Confirmatory factor analysis results

The structure of the ACEM–Impact was assessed using inter-item correlations and a series of factor analysis models before fitting the final confirmatory factor analysis models. Model fit statistics, correlation residuals and qualitative input were considered. The final structure was defined considering both these data results and review of these analyses and the qualitative concept elicitation development results. For example, the final framework removed the assistance/adaptive devices items because upon review they were perceived as more distal outcomes rather than indicators of ACEM–Impact.

The final proposed structure for the ACEM–Impact scale split the 13-item Functioning domain into three separate domains corresponding to Daily Living Functioning, Physical Functioning and School Functioning. The 7-item Social Well-being domain was split into a separate 3-item Social Functioning and 3-item Social Well-being domain with one item “being treated as younger than they are” dropped from the scoring and conceptual framework. This decision was made, in part, since some children who were interviewed reported benefit from being treated as younger and others reported negative impact. Thus, the broad nature of the concepts reflected in the original structure were refined in the final structure into domains that reflected more discreet and interpretable areas of impact. Finally, the conceptual framework to define scoring removed the assistance/adaptive devices items because these were perceived as more distal outcomes rather than indicators of ACEM–Impact.

The revised ACEM–Impact conceptual framework consisted of 25 items that cluster into six domains. The remaining items were not included in an ACEM–Impact summary score but are retained in the questionnaire to be used as limited item-level descriptive statistics. Thus, the final domains of the measure were Daily Living Functioning, Physical Functioning, School, Social Functioning, Social Well-being, and Emotional Well-being. Supplementary file 3 presents the ACEM–Impact item-level development summary for each item including the original hypothesized conceptual framework and the final conceptual framework.

The revised 6-domain ACEM–Impact model was fit to 25 impact items. The estimated standardized factor loadings, inter-factor correlations, and key fit statistics are displayed in Table 5. The items within each domain were determined to be adequately associated with the domains, as all factor loadings were 0.60 or greater. Although the fit statistics did not meet all prespecified criteria (CFI = 0.90; TLI = 0.89; RMSEA = 0.11), the magnitude of the factor loadings and the expected pattern in the inter-factor correlations supported the final proposed structure.

**Table 5:** Six-factor CFA model for ACEM–Impact, baseline

Domain/Item	Standardized Estimates (Standard Errors)					
	Factor 1	Factor 2	Factor 3	Factor 4	Factor 5	Factor 6
**Daily Living Functioning domain**						
Reach objects or high places	0.601* (0.048)	—	—	—	—	—
Toilet themselves	0.750* (0.029)	—	—	—	—	—
Bathe/shower, wash, and/or comb hair	0.844* (0.024)	—	—	—	—	—
Perform tasks requiring fine motor skills	0.818* (0.037)	—	—	—	—	—
Dress or undress	0.875* (0.026)	—	—	—	—	—
**Physical Functioning domain**						
Sit for long periods of time	—	0.759* (0.034)	—	—	—	—
Climb stair or steps	—	0.805* (0.029)	—	—	—	—
Be physically active	—	0.881* (0.025)	—	—	—	—
Walk long distances	—	0.747* (0.029)	—	—	—	—
Run	—	0.860* (0.021)	—	—	—	—
**School domain**						
Have difficulty participating in class/schoolwork activities	—	—	0.866* (0.033)	—	—	—
Limit or modify their participation in physical education (gym class)	—	—	0.784* (0.051)	—	—	—
Miss school/preschool	—	—	0.688* (0.047)	—	—	—
**Emotional Well-being domain**						
Different from others	—	—	—	0.819* (0.026)	—	—
Frustrated	—	—	—	0.864* (0.024)	—	—
Angry	—	—	—	0.859* (0.022)	—	—
Sad	—	—	—	0.879* (0.019)	—	—
Worried	—	—	—	0.915* (0.016)	—	—
Embarrassed	—	—	—	0.859* (0.023)	—	—
**Social Functioning domain**						
Physically keep up with other children their age	—	—	—	—	0.794* (0.034)	—
Participate in social activities	—	—	—	—	0.884* (0.030)	—
Participate in sports or physical play	—	—	—	—	0.804* (0.029)	—
**Social Well-being domain**						
Teasing/bullying	—	—	—	—	—	0.900* (0.023)
Negative attention in public	—	—	—	—	—	0.760* (0.037)
Being stigmatized	—	—	—	—	—	0.843* (0.030)
**Factor correlations**						
**Factor 2**	0.703* (0.036)	—	—	—	—	—
**Factor 3**	0.596* (0.068)	0.784* (0.053)	—	—	—	—
**Factor 4**	0.210* (0.064)	0.577* (0.048)	0.827* (0.043)	—	—	—
**Factor 5**	0.588* (0.052)	0.743* (0.041)	0.852* (0.046)	0.631* (0.051)	—	—
**Factor 6**	0.218* (0.067)	0.639* (0.045)	0.718* (0.052)	0.847* (0.031)	0.653* (0.054)	—
**Fit statistics**						
**Chi-square**, ***df***, ***P***	914.574, 260, < 0.001					
**RMSEA**	0.112					
**CFI**	0.902					
**TLI**	0.887					
**SRMR**	0.084					

ACEM–Impact = Achondroplasia Child Experience Measure-Impact; CFA = confirmatory factor analysis; CFI = comparative fit index; df = degrees of freedom; RMSEA = root mean square error of approximation; SRMR = standardized root mean residual; TLI = Tucker-Lewis index

#### Scale-level internal consistency reliabilities

The internal consistency reliabilities of the total and domain scores were strong, ranging from 0.76 to 0.93 (*n* = 107–197, observational data) (Supplementary file 2, Table S4).

#### Convergent/divergent validity

For the observational study assessment, the findings supported the hypothesized relationships. Specifically, ACEM–Impact Total scores correlated strongly with all PGIS-Child Impact items (ranging from *r* = 0.57 with PGIS-Child Impact Adaptive devices or assistance item to *r* = 0.76 with PGIS-Child Impact Quality of life item; strongly with all QoLISSY scores, except QoLISSY Coping (*r* = 0.02); and strongly with all GHD-CIM scores, except for a moderate correlation with GHD-CIM Physical Functioning (*r* = 0.39).

In the phase 2 interventional trial data, the predicted correlations were moderate to strong, with a few exceptions. ACEM–Impact Total scores correlated moderately to strongly with all GHD-CIM scores, except for a small correlation with GHD-CIM Emotional Well-being (screening = 0.28); moderately to strongly with all QoLISSY scores except for QoLISSY General beliefs about height (screening = − 0.24); and moderately to strongly with all PGIS-Child Impact ratings except PGIS-Child Impact Emotional Well-being (screening = 0.28).

Based on these findings, convergent validity evidence for the ACEM–Impact Total and domain scores can be considered adequate (Supplementary file 2, Table S5).

#### Known-groups validity

Known groups validity for both the observational and the interventional trial was satisfactory with at least one hypothesis per domain and the total score met. Additionally, post-hoc analyses were conducted to look at the influence of age on the total score. Analyses found that there was not a significant difference between children aged less than 5 years and 5 years or older (Supplementary file 2, Table S6).

#### Test-retest reliability

Test-retest results were above the 0.7 recommended minimum threshold for all three subgroup evaluations for majority of the domains and within the 95% confidence interval for all scores providing support for adequate test-retest reliability (Supplementary file 2, Table S7).

#### Ability to detect change

Table 6 displays the descriptive statistics of change scores by levels of the corresponding PGIS item used as the external criterion for change. Using the PGIS items as external criteria, the mean differences between PGIS improved group versus the PGIS no change/worsening group were statistically significant for the ACEM–Impact Emotional Well-being (by PGIS-Child Impact Emotional item) and Daily Living Functioning (*P* ≤ 0.05). Although not statistically significant for the other ACEM–Impact scores, the mean differences were in the correct direction indicating a trend such that participants who reported improvement in terms of PGIS ratings also showed improvement on the corresponding ACEM–Impact scores.

**Table 6:** ACEM–Impact ability to detect change

Transformed ACEM–Impact score	Improved group		No change/worsening group		t-statistic (*P* value)
	*n*	LS mean (SE), median, min, max, (95% CI)	*n*	LS mean (SE), median, min, max 95% CI)	
ACEM–Impact Total by PGIS-Child Impact Quality of life item	12	−4.57 (3.8), − 4.6, − 34.7, 23.8, (− 14.06–4.92)	25	−0.19 (2.6), − 1.7, − 18.6, 32.9, (− 5.22–4.84)	−0.95 (0.35)
ACEM–Impact Daily Living Functioning by PGIS-Child Impact Physical functioning item	19	−7.42 (4.4), − 8.0, − 37.0, 32.0, (− 15.37–0.53)	17	−3.35 (4.6), − 8.0, − 40.0, 40.0, (− 14.43–7.72)	−0.64 (0.53)
ACEM–Impact Physical Functioning by PGIS-Child Impact Physical functioning item	19	−8.21 (3.1), − 8.0, − 44.0, 20.0, (− 14.93 to − 1.49)	18	0.67 (3.2), 0.0, − 20.0, 28.0, (− 5.78–7.11)	−2.00 (0.05)
ACEM–Impact School by PGIS-Child Impact Physical functioning item	8	−6.25 (6.0), − 4.2, − 25.0, 0.0, (− 13.46–0.96)	11	9.85 (5.2), 8.3, − 25.0, 50.0, (− 4.33–24.03)	−2.03 (0.06)
ACEM–Impact Emotional Well-being by PGIS-Child Impact Emotional item	9	−8.33 (5.1), 0.0, − 45.8, 20.8, (− 25.66–8.99)	21	5.56 (3.4), 4.2, − 12.5, 33.3, (0.42–10.69)	−2.27 (0.03)
ACEM–Impact Social Well-being by PGIS-Child Impact Interactions with peers item	7	3.57 (5.3), 0.0, − 33.3, 25.0, (− 17.70-24.84)	25	5.33 (2.8), 0.0, − 16.7, 33.3, (1.02–9.65)	−0.30 (0.77)
ACEM–Impact Social Functioning by PGIS-Child Impact Limitations with friends item	16	−3.91 (6.0), − 8.3, − 41.7, 50.0, (− 15.01–7.19)	21	2.78 (5.3), 0.0, − 41.7, 58.3, (− 9.22–14.78)	−0.83 (0.41)

ACEM-Impact = Achondroplasia Child Experience Measure-Impact; CI = confidence interval; LS = least squares; PGIS = patient global impression of severity; SE = standard error

### Conceptual frameworks

**Fig. 1:**
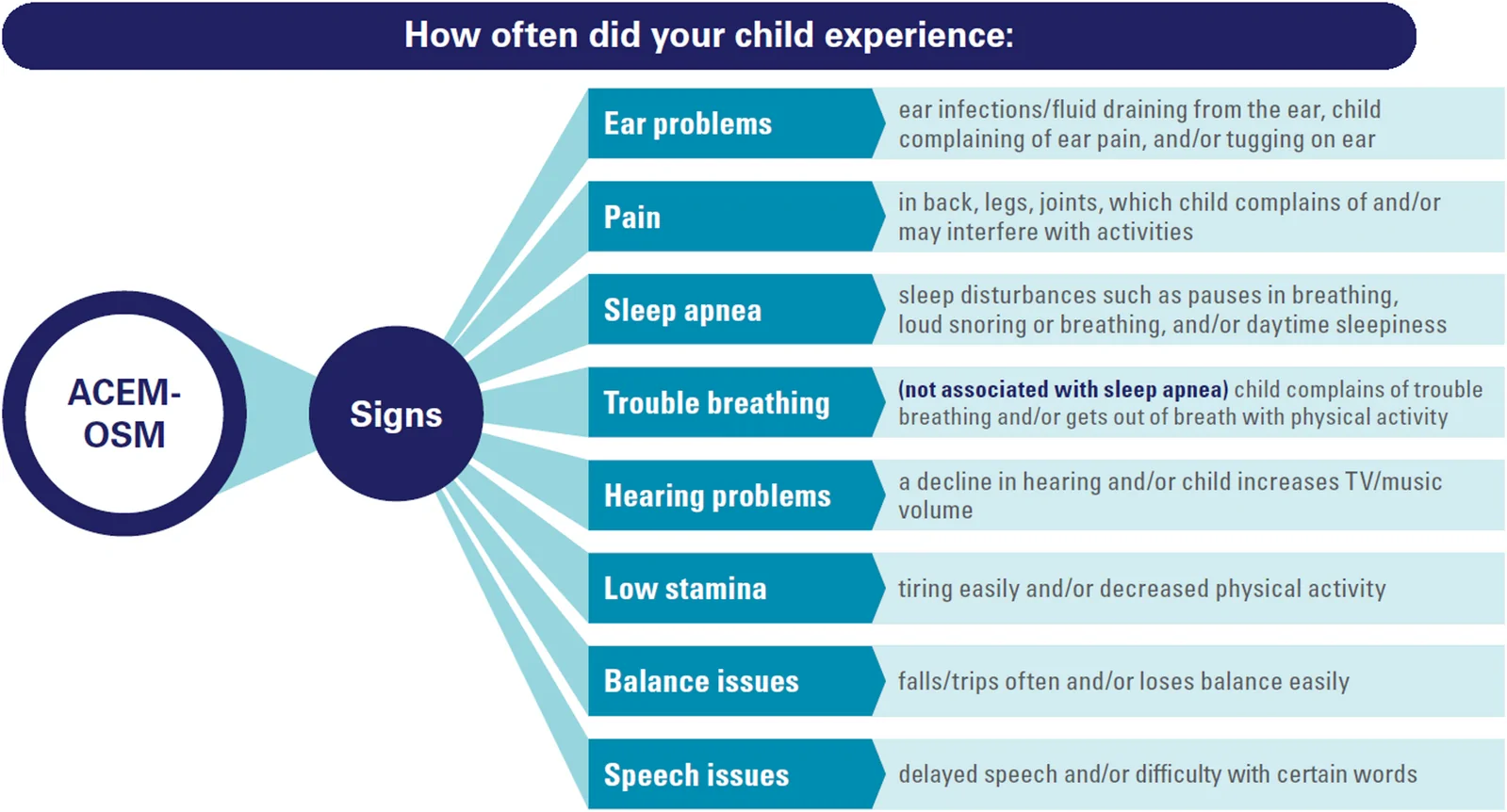
Final conceptual framework of the ACEM-OSM; ACEM–OSM = Achondroplasia Child Experience Measure–Observable Signs Measure

**Fig. 2:**
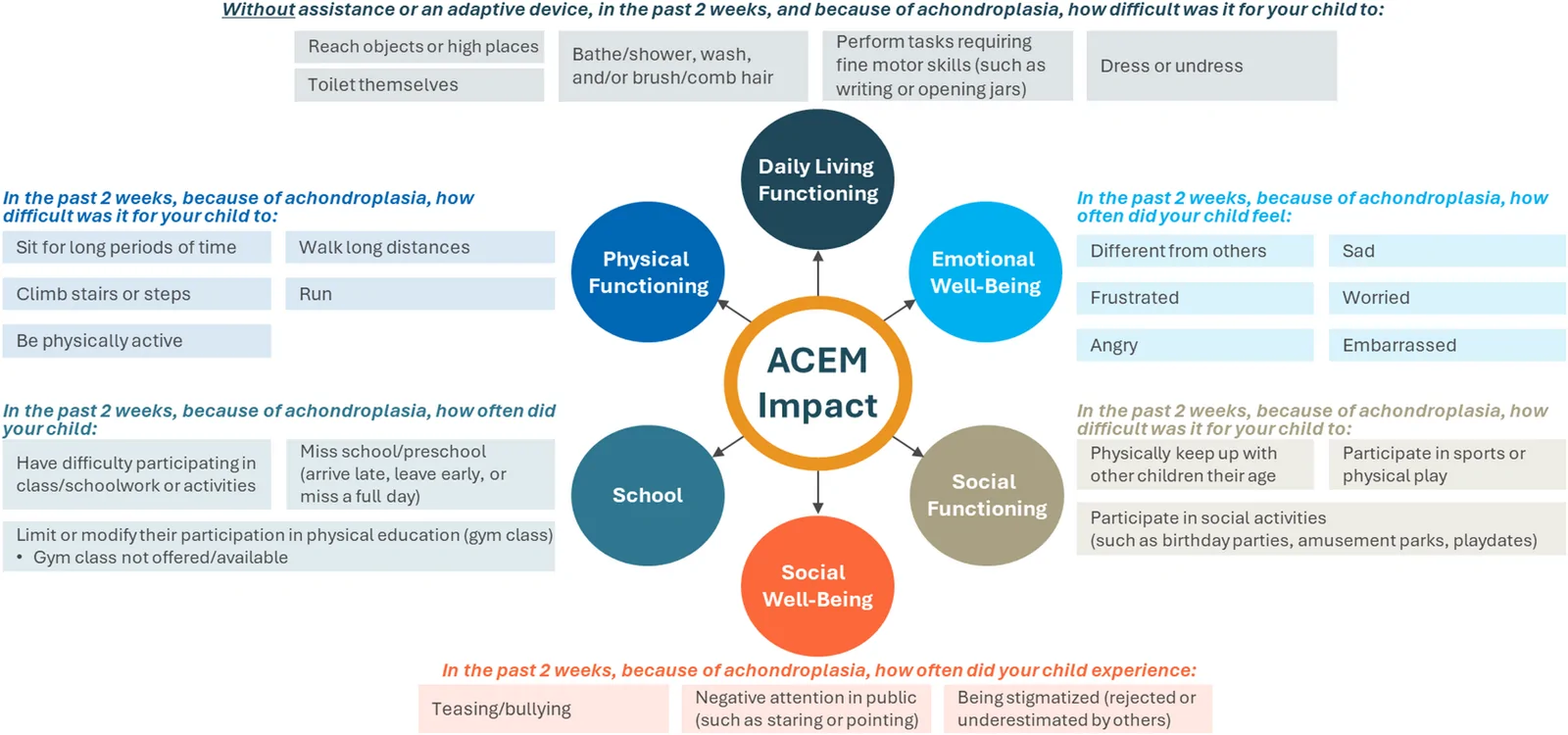
Final ACEM–Impact conceptual framework; ACEM-Impact = Achondroplasia Child Experience Measure-Impact

Based on the evaluation results, the conceptual frameworks of the final, validated ACEM–OSM and ACEM–Impact are shown below (Figs. 1 and 2).

## Discussion

The present study sought to develop methodologically sound, condition-specific measures for achondroplasia in children. The ACEM–OSM and the ACEM–Impact are novel measures designed to capture the broad spectrum of the achondroplasia experience in children. The measures are developed according to well-defined scientific principles for the development of PROs and ObsROs and evaluated in accordance with recommendations outlined in the FDA PRO guidance.

This study sought to build upon the work of others in the field of achondroplasia in order to create observer-reported measures of the impact of the condition on children. These new measures were needed as existing measures were evaluated to be not optimal as condition-specific clinical outcome assessments. For example, QoLISSY is not condition-specific and focuses only on short stature. Similarly, the Child Health Assessment Questionnaire (CHAQ) [43] is not specific to achondroplasia. To measure the impact of the condition, researchers need a measure that is condiiton-specific [19]. While APLES is the closest fit, it was not developed in accordance with FDA guidelines in that the authors did not fully consider the patient perspective, therefore, limiting its content validity [18]. Further, the APLES is appropriate for children starting with age 5 years. Thus, the ACEM–OSM and ACEM–Impact fill a critical need for measures that can capture the broad spectrum of achondroplasia experiences in children and includes children starting at age 2.

Review of the item-level responses indicated that the interventional trial sample represented a milder sample (higher ceiling effects) than the observational sample, with a large proportion of responses at the best response category. This finding is likely due to the inclusion criterion that the child must be able to stand without assistance, suggesting a less impaired population. Thus, the sample was relatively mild (i.e., not severe), which may have contributed to ceiling effects and limited responsiveness findings. Further, the relatively small size of the interventional trial (*N* = 57) may have influenced some of the non-significant findings for ability to detect change. However, results from both samples provided evidence in support of reliability, cross-sectional construct validity, and an initial exploration of the ability to detect change and the consistency across multiple studies are strengths of this evaluation.

It should be noted that some analyses required data to be available at two time points, thus adding some variability to sample sizes across analyses. For example, the school-related items are only applicable if the child attended school or preschool within the recall period. For the test-retest reliability, this would require the child to have attended school or preschool at both time points. Despite this variability, the psychometric findings were shown to be acceptable. Additionally, as this sample included both children of parents with achondroplasia as well as of average height parents, the ACEMs can be considered appropriate for all children with achondroplasia, regardless of the achondroplasia status of their parents.

It is our hope that the ACEMs will be used in future studies of new treatments. When interpreting results obtained from the ACEMs, it is important to consider the duration of the study in which these assessments are used. Many treatment-related effects take time to manifest, and short-term evaluations may not capture the full extent of meaningful change across relevant outcomes. Further, in interpreting results from these measures, the baseline severity of the achondroplasia (as assessed by such criteria as ability to walk without aids, baseline height), lower limb malalignment, disproportionality as well as other factors such as access to resources, should be taken into consideration as this may influence outcomes.

It is also important to recognize that outcomes do not speak to etiology of change captured by the measures. This is especially important for achondroplasia, where the specific mechanisms by which functional improvements occur are unclear and height, often the primary outcome of choice for regulatory approval of new treatments, has been shown to not be the most important result of treatment by parents and children diagnosed with achondroplasia [13]. The landscape of achondroplasia and its treatment is changing with the introduction of new treatments and the potential of treatments under development with differing mechanisms of action. Unfortunately, when the observational study was launched, there was no approved pharmaceutical treatment in children with achondroplasia. In the future, careful assessment of the effects of new treatments should assess meaningful change for a wide range of clinical features, beyond growth, as well as impacts on daily life. Additionally, clinicians who treat these children should see benefit by incorporating the ACEMs into their treatment plans to help inform them of the full range of symptoms and impacts important to this population. Discussion with patients and their families about what matters to them along with changes seen through ACEM scores will hopefully lead to improved provider-patient relationships and adherence to treatments.

The timing of treatment initiation can also influence outcomes as assessed by the ACEMs, especially the ACEM-Impact. The ACEMs are appropriate for children beginning at age 2. It is evident that younger children do not have the same social and psychological awareness as older children, which develops once they start interacting more with their peers and attending nursery or school [44]. A parent burden measure may be more appropriate for this young age group, as it is the parent who carries the responsibility for managing the condition. Further, children at different stages of development have different needs both socially and physically. Therefore, depending upon at what age treatment is initiated, different aspects of their lives may be more affected than others. Based on data from this study, the ACEMs are appropriate for children aged 2 to < 12 years, captures signs and impacts that are relevant across this age span, and can be considered relevant for this age group. It should also be noted that the ACEMs were developed on children who have already experienced up to 12 years of living with achondroplasia untreated. It is unclear how this prior experience has shaped the impacts experienced in the present or how treatment early in life may impact future symptoms and impacts.

Some limitations of this study should be noted. First, children with limb lengthening surgery were excluded from the sample. It would be important to look at how surgery impacts these outcomes. Future research should also examine differences between children with average height parents vs. those with parents with achondroplasia as well as define what constitutes meaningful change in these measures. Second, CFA model fit results for ACEM–Impact fell below the prespecified thresholds for acceptable fit. However, the results did provide information along with the qualitative development data to guide selection of the final proposed structure grouped aspects of impact which maintained the patient centric focus of the final measure thus, facilitating appropriate interpretation of scores. Third, without anchor-based or distribution-based minimum clinically important difference estimates, it is difficult to interpret what a meaningful score change looks like. Therefore, it is recommended that there be additional assessments of meaningful change in future studies to build evidence in support of preliminary threshold estimates. Additionally, sample sizes in the interventional study may not have been adequate to detect meaningful change. Although not statistically significant for some of the ACEM–Impact scores, the mean differences were in the correct direction indicating a trend such that participants who reported improvement in terms of PGIS ratings also showed improvement on the corresponding ACEM–Impact scores.

Finally, this study was conducted in the US and Spain for concept elicitation and in 8 countries across multiple continents for the validation study. Cultural influences may play a part in how achondroplasia is perceived by the society and accommodations made, which may affect outcomes. Further research is needed to better understand these influences. For example, future research should be conducted on larger, heterogenous samples to further test these measures [18].

The present study shows that the ACEMs are reliable and valid measures of the signs and impacts of achondroplasia in children. Incorporation of these measures in clinical and research settings can aid in assessing new treatments and improve our understanding of the experience of those living with achondroplasia. For instance, these new measures can help determine if improvement in clinical outcomes are meaningful and also allow clinicians to examine the overall impact of achondroplasia on children, beyond height.

## Conclusions

In conclusion, the ACEMs are well developed, reliable and valid measures of the signs and impacts of achondroplasia in children aged 2 to < 12 years. Incorporation of these measures in both clinical and research settings can help move the field forward in assessing new treatments and improving our understanding of the experience of those living with achondroplasia.

## Supplementary Information

Below is the link to the electronic supplementary material.


Supplementary Material 1



Supplementary Material 2



Supplementary Material 3


## Data Availability

The datasets used and/or analyzed for the research presented in the publication may be available on a case-by-case basis for reasonable requests from the corresponding author.

## Abbreviations

ACEM: Achondroplasia Child Experience Measure
ACEM–Impact: Achondroplasia Child Experience Measure-Impact
ACEM–OSM: Achondroplasia Child Experience Measure-Observable Signs Measure
ACH: Achondroplasia
ANOVA: Analyses of variance
CFA: Confirmatory factor analysis
CFI: Comparative fit index
CI: Confidence interval
DCGM: DISABKIDS Chronic Generic Module (DCGM-37) Proxy version
df: Degrees of freedom
FDA: United States Food and Drug Administration
FGFR3: Fibroblast growth factor receptor 3
GHD-CIM: Growth Hormone Deficiency Child Impact Measure
ICC: Intraclass correlation coefficient
IT: Interventional trial
IRB: Institutional Review Board
LS: Least squares
ObsRO: Observer-reported outcome
OS: Observational study
PedsQL: Pediatric Quality of Life Inventory
PGIS: Patient Global Impression of Severity
PRO: Patient-reported outcome
PROMIS: Patient-Reported Outcomes Measurement Information System
QoLISSY: Quality of Life in Short Stature Youth
RMSEA: Root mean square error of approximation
SD: Standard deviation
SE: Standard error
SF-10: 10-item Short Form Health Survey for Children (proxy)
SRMR: Standardized root mean residual
SWLS-C (proxy): Satisfaction with Life Scale – Child (proxy)
TLI: Tucker-Lewis index
US: United States
