# Supplementary material for: Assessing signs and impacts of achondroplasia: psychometric evaluation of the Achondroplasia Child Experience Measures
Source: J Patient Rep Outcomes. 2026 Jun 19;10:162. doi: 10.1186/s41687-026-01123-z (PMC13534396; doi:10.1186/s41687-026-01123-z)
Supplement: Supplementary file 1 [file 41687_2026_1123_MOESM1_ESM.docx]

Supplementary file 1

**Assessing Signs and Impacts of Achondroplasia: Psychometric Evaluation of the Achondroplasia Child Experience Measures**

Table A contains details about the supporting measures used in the PRO/ObsRO Validation Battery in addition to the Achondroplasia Child Experience Measures (ACEMs), presented in the order of administration to the parent/guardian.

Table A. PRO/ObsRO Validation Battery of Self-Administered Measures

| Validated Measures^[1]^ | Measure description |
| --- | --- |
| Patient-Reported Outcomes Measurement Information System (PROMIS) – Parent Proxy [Irwin, 2012; Forrest, 2014] | The child’s primary caregiver answered PROMIS measures to capture their proxy assessment over the past two weeks of the child’s physical, mental, and social health using the: *Global Health* 7 + 2-item measure assessing a child’s physical, mental, and social health, plus pain and fatigue (in general and in the past 2 weeks); *Upper Extremity* short form 8-item measure assessing a child’s ability to conduct activities with their upper extremities, including shoulder, arm, and hand activities; and *Depressive Symptoms* short form 6-item measure. |
| Satisfaction with Life Scale – Child (SWLS-C) (proxy) [Gadermann, 2010] | The SWLS-C is a 5-item measure of a child’s satisfaction with his or her life as reported by their parent/guardian. |
| Pediatric Quality of Life Inventory (PedsQL™) 4.0 Generic Core Scales – Parent version [Varni, 2001] | The PedsQL measures parents’/guardians’ assessment of the HRQoL of their child with ACH over the past two weeks in the areas of physical functioning, emotional functioning, social functioning, and school functioning. The parent answered an age-appropriate version based upon the age of their child at the time of the assessment [age groups: 2-4 years (21 items), 5-7 years (23 items), and 8 years or greater (23 items)]. |
| Short Form Health Survey (SF-10) for Children (proxy); acute version [Saris-Baglama, 2007] | The SF-10 for Children 10-item acute version (over the past week) is a generic measure of perceived health status (child’s health and well-being) , as assessed by the child’s parent/guardian, in the areas of physical functioning, role limitations due to physical health problems, bodily pain, social functioning, general mental health, role limitations due to emotional problems, vitality, and general health perceptions. |
| Growth Hormone Deficiency Child Impact Measure (GHD-CIM) – ObsRO version [Brod, 2021] | The 11-item GHD-CIM ObsRO version was adapted to assess the impact of ACH on the child’s life, over the past two weeks, in the areas of physical functioning, social well-being, and emotional well-being. The parent/guardian was instructed to answer based upon what they had seen or been told by their child or by others about their child. |
| DISABKIDS Chronic Generic Module (DCGM-37); Proxy version [Bullinger, 2002; Petersen, 2005] | The DISABKIDS (DCGM) is a generic questionnaire assessing the HRQoL of children and adolescents with chronic medical conditions from their parents’ or caregivers’ perspectives. Respondents were instructed to think about how their child was feeling over the past 2 weeks related to their child’s ACH when answering the questions*.* For the study, 19 items were administered from the Independence, Emotion, and Limitation subscales. |
| Quality of Life in Short Stature Youth (QoLISSY) [Bullinger, 2013) | 52-items of the QoLISSY measure were administered to evaluate the impact of short stature on children and the parent based on the primary caregiver’s perspective. The parent was instructed to answer how their child feels related to his/her height, over the past two weeks, in the areas of physical, social, emotional aspects of functioning and well-being, as well as in the areas of coping, general beliefs about height, and belief about (child’s) future. |

The PRO/ObsRO battery used in both the non-interventional study, and the phase 2 clinical trial also included items to capture:

- **Socio-demographic items** (e.g., gender, race, ethnicity, age) for child and parent/guardian; child’s medical (achondroplasia) history.
- Proxy-reported **Patient Global Impression of Severity (PGIS)** to align with the ACEM-OSM domain; and proxy-reported Overall Impact of achondroplasia on the child’s QoL, physical functioning, emotional well-being, social well-being, and use of adaptive devices/need for assistance to align with the ACEM-Impact domains.
- **Major Life Changes** (e.g., experiencing a move or relocation, divorce, school or job change, birth, or the adoption of a child) experienced by the child and/or parent (retest) in the past 2 weeks (yes/no).

The PRO/ObsRO battery used in the non-interventional study included Resource Use related to child’s achondroplasia such as out-of-pocket health care expenses and money spent on modifications, devices, and equipment. Additional observational study items included those relevant to the data being collected such as a single question to record if the child was currently enrolled, or expected to enroll in the next three months, in an achondroplasia treatment clinical trial, and questions about any potential impacts on the child due to the COVID-19 pandemic in the areas of activities of daily life, work/school, or physical, emotional, and social functioning.

The PRO/ObsRO battery used in the phase 2 clinical trial included items relevant to the psychometric evaluation of the measures such as a Clinician Global Impression of Severity (CGI-S) item and clinician measurements of annualized height velocity (AHV).

1. For validated measures, when applicable and permitted by measure developers, minor adaptations were made to the align the recall period with “over the past 2 weeks” of the ACEMs and to revise condition-specific language to be relevant to achondroplasia.
