# Supplementary material for: Assessing signs and impacts of achondroplasia: psychometric evaluation of the Achondroplasia Child Experience Measures
Source: J Patient Rep Outcomes. 2026 Jun 19;10:162. doi: 10.1186/s41687-026-01123-z (PMC13534396; doi:10.1186/s41687-026-01123-z)
Supplement: Supplementary file 2 [file 41687_2026_1123_MOESM2_ESM.docx]

**Supplementary File 2**

**Assessing Signs and Impacts of Achondroplasia: Psychometric Evaluation of the Achondroplasia Child Experience Measures**

**Table S1. ACEM–OSM Inter-item and Item-total Correlations, Baseline (n = 142 to 148)**

| ACEM-OSM Item | 1 | 2 | 3 | 4 | 5 | 6 | 7 | 8 |
| --- | --- | --- | --- | --- | --- | --- | --- | --- |
| 1 Ear problems | — |  |  |  |  |  |  |  |
| 2 Pain | 0.48* | — |  |  |  |  |  |  |
| 3 Sleep apnea | 0.47* | 0.48* | — |  |  |  |  |  |
| 4 Trouble breathing | 0.64* | 0.53* | 0.57* | — |  |  |  |  |
| 5 Hearing problems | 0.63* | 0.54* | 0.63* | 0.75* | — |  |  |  |
| 6 Low stamina | 0.52* | 0.48* | 0.44* | 0.54* | 0.52* | — |  |  |
| 7 Balance issues | 0.47* | 0.47* | 0.41* | 0.63* | 0.49* | 0.47* | — |  |
| 8 Speech issues | 0.40* | 0.32* | 0.58* | 0.66* | 0.62* | 0.42* | 0.46* | — |
| Item-total correlation | 0.62 | 0.57 | 0.65 | 0.79 | 0.74 | 0.58 | 0.60 | 0.62 |

ACEM–OSM = Achondroplasia Child Experience Measure-Observable Signs Measure

**Table S2. ACEM–OSM Known-Groups Validity Results Summary**

| **ACEM Measure** | **Supporting Measure** | **Results** | | | **Hypothesis** |
| --- | --- | --- | --- | --- | --- |
|  |  | **IT: Screening** | **IT: Visit 7** | **OS: Baseline (Adapted Version)** |  |
| ACEM-OSM Total score | SF-10 global health item |  Overall test *P* < 0.01, means properly ordered |  Overall test *P* < 0.01, means properly ordered |  Overall test *P* < 0.01, means properly ordered except “Good” | Caregivers with better SF-10 Global Health item scores will have lower mean ACEM–OSM scores than caregivers with worse SF-10 Global Health item scores. |
|  | PedsQL domains (including Psychosocial Health Summary) and Total |  Overall tests P < 0.05 for 3 of the 5 domains and the Total Score   Means properly ordered except for Psychosocial Health Summary |  Overall tests *P* < 0.01, except for “Social Functioning”; means properly ordered except for “School Functioning” |  All 6 overall tests *P* < 0.01, means properly ordered | Caregivers with better PedsQL domain scores will have lower mean ACEM–OSM scores than caregivers with worse PedsQL scores. |
|  | PGIS-Child Symptom |  Overall test *P* < 0.01, means properly ordered |  Overall test *P* < 0.01, means properly ordered |  Overall test *P* < 0.01, means properly ordered | Caregivers with better PGIS-Child Symptom ratings will have lower mean ACEM–OSM scores than caregivers with worse PGIS-Child Symptom ratings. |

ACEM–OSM = Achondroplasia Child Experience Measure-Observable Signs Measure; IT = interventional trial; PedsQL = Pediatric Quality of Life Inventory 4.0 Generic Core Scales – Parent version; OS = observational study; PGIS = parent global impression of severity; SF-10 = 10-item Short Form Health Survey for Children (proxy).

Note: Mean comparisons were not conducted for groups with n < 5. SF-10 Global Health item and PGIS-Child Symptom rating subgroups were defined by the individual item response categories.

**Table S3. ACEM–OSM Test-Retest Reliability**

**Test-Retest Reliability, Baseline to Retest (Week 2), observational study**

|  | **Overall ICC (95% CI), n** | **No major life change ICC (95% CI), n** | **No change in PGIS ICC (95% CI), n** |
| --- | --- | --- | --- |
| **ACEM**–**OSM** | 0.73 (0.64-0.80), 131 | 0.70 (0.58-0.79), 92 | 0.76 (0.63-0.85), 60 |

| Test-Retest Reliability, interventional trial | | | | |
| --- | --- | --- | --- | --- |
|  | Screening to baseline Overall ICC (95% CI), n | Screening to baseline No major life change ICC (95% CI), n | Visit 3 to visit 4 Overall ICC (95% CI), n | Visit 3 to visit 4 No change in PGIS ICC (95% CI), n |
| **ACEM-OSM** | 0.88 (0.78-0.94), 36 | 0.88 (0.78-0.94), 34 | 0.75 (0.59-0.86), 44 | 0.70 (0.43-0.86), 24 |

ACEM–OSM = Achondroplasia Child Experience Measure-Observable Signs Measure; CI = confidence interval; ICC = intraclass correlation coefficient; PGIS = parent global impression of severity.

**Table S4. ACEM**–**Impact Internal Consistencies, Baseline**

|  | **Cronbach’s alpha, n** |
| --- | --- |
| **ACEM**–**Impact Total** | 0.86, 122 |
| Daily Living Functioning | 0.85, 197 |
| Physical Functioning | 0.88, 194 |
| School | 0.76, 107 |
| Emotional Well-being | 0.93, 183 |
| Social Functioning | 0.83, 193 |
| Social Well-being | 0.84, 183 |

ACEM–Impact = Achondroplasia Child Experience Measure-Impact

**Table S5. ACEM–Impact Convergent and Divergent Validity Evidence (Observational Study Baseline; Interventional Trial Screening and Visit 7)**

| **Measure** | **Supporting Measure** | Construct Validity Results |
| --- | --- | --- |
| ACEM–Impact Total score | GHD-CIM domains | **✓** OS: Moderate to strong correlations with all 4 GHD-CIM domains (convergent validity); IT: moderate to strong correlations with 3 of 4 GHD-CIM domains at both timepoints |
|  | QoLISSY domains | **✓** OS: Strong correlations with 7 of 8 QoLISSY domains (convergent validity); IT: moderate to strong correlations with 7 of 8 QoLISSY domains at both timepoints |
|  | PGIS‑Child Impact items | **✓** OS: Strong correlations with all 7 PGIS‑Child Impact ratings (convergent validity); IT: moderate to strong correlations with most PGIS ratings at both timepoints |
| ACEM–Impact Functioning score | DCGM Physical | **✓** OS: Moderate correlation (r = −0.49) with DCGM Physical Limitations (convergent validity) and lower correlations with the other DCGM domains (divergent validity); IT: moderate to strong correlations at both timepoints (r_screening_ = −0.49, r_visit 7_ = −0.50) |
|  | PedsQL Physical Functioning | **✓** OS: Strong correlation (r = −0.68) with PedsQL Physical Functioning (convergent validity) and lower correlations with all other PedsQL domains (divergent validity); I: strong correlations with PedsQL Physical Functioning scores at both timepoints (r_screening_ = −0.72, r_visit 7_ = -0.68) |
|  | PGIS‑Child Impact  Physical functioning item | **✓** OS: Strong correlation (r = 0.59) with PGIS‑Child Impact Physical Functioning (convergent validity), with some lower correlations with the other PGIS items (divergent validity); IT: strong correlations with PGIS‑Child Impact Physical Functioning at both timepoints (r_screening_ = 0.67, r_visit 7_ = 0.75) |
| ACEM–Impact Daily Living Functioning score | GHD-CIM domains | OS: Small to trivial correlations with all 4 GHD-CIM domains; IT: moderate to strong correlations at both timepoints with 3 of 4 GHD-CIM domains (convergent validity) |
|  | QoLISSY domains | OS: Moderate correlation with QoLISSY Physical Core, small to trivial correlations with 7 of 8 QoLISSY domains; IT: strong correlations with QoLISSY Physical Core at both timepoints (r_screening_ = −0.68, r_visit 7_ = −0.62) (convergent validity) |
|  | PGIS‑Child Impact items | OS: Moderate correlations with 3 PGIS‑Child Impact ratings, small to trivial correlations with 4 PGIS‑Child Impact ratings; IT: moderate to strong correlations at both timepoints with 5 of 7 PGIS‑Child Impact ratings (convergent validity) |
| ACEM–Impact Physical Functioning score | GHD-CIM domains | **✓** OS: Moderate to strong correlations with all 4 GHD-CIM domains (convergent validity); IT: moderate to strong correlations at both timepoints with 3 of 4 GHD-CIM domains |
|  | QoLISSY domains | **✓** OS: Moderate correlations with 7 of 8 QoLISSY domains (convergent validity); IT: moderate to strong correlations at both timepoints with 5 of 8 QoLISSY domains |

ACEM-Impact = Achondroplasia Child Experience Measure-Impact; DCGM = DISABKIDS Chronic Generic Module (DCGM-37) Proxy version [38,39]; GHD-CIM = Growth Hormone Deficiency Child Impact Measure ObsRO version; IT = interventional trial; PedsQL = Pediatric Quality of Life Inventory 4.0 Generic Core Scales – Parent version; PGIS = patient global impression of severity; QoLISSY = Quality of Life in Short Stature Youth.

**Table S6. ACEM–Impact Known-Groups Validity Results Summary**

| **Measure** | **Supporting Measure** | **Results** | | | | | **Hypothesis** |
| --- | --- | --- | --- | --- | --- | --- | --- |
|  |  | **IT: Screening** | | **IT: Visit 7** | **OS: Baseline** | |  |
| ACEM–Impact Total score | SF-10 Physical Summary Score | ✓ Overall test *P* < 0.05, means properly ordered | ✓ Overall test *P* < 0.01, means properly ordered | | | ✓ Overall tests *P* < 0.01, means properly ordered | Caregivers with better SF-10 domain scores will have lower ACEM–Impact Total scores than caregivers with worse SF-10 scores. |
|  | PedsQL Physical domain | ✓ Overall test *P* < 0.01, means properly ordered | ✓ Overall test *P* < 0.01, means properly ordered | | | ✓ Overall tests *P* < 0.01  ✓ Means properly ordered | Caregivers with better PedsQL domain scores will have lower mean ACEM–Impact Total scores than caregivers with worse PedsQL scores. |
| ACEM-Impact Physical Functioning score | PROMIS Parent Proxy Upper Extremity SF-8a | Overall test *P* = 0.35, means properly ordered | **✓** Overall test *P* < 0.05, means properly ordered | | | **✓** Overall tests *P* < 0.01, means properly ordered | Caregivers who report greater upper extremity interference will have higher (worse) mean ACEM-Impact Physical Functioning scores than caregivers who report less interference. |
| ACEM-Impact Daily Living Functioning | PROMIS Parent Proxy Upper Extremity SF-8a | **✓** Overall test *P* < 0.01, means properly ordered | **✓** Overall test *P* < 0.01, means properly ordered | | | **✓** Overall test *P* < 0.01, means properly ordered | Caregivers who report greater upper extremity interference will have higher mean ACEM-Impact Daily Living Functioning scores than caregivers who reported less interference. |
| ACEM-Impact School score | PROMIS Parent Proxy Upper Extremity SF-8a | Overall test *P* = 0.44 | Overall test *P* = 0.20, means properly ordered | | | **✓** Overall test *P* < 0.01, means properly ordered | Caregivers who report greater upper extremity interference will have higher (worse) mean ACEM-Impact School scores than caregivers who report less interference (OS only). |
| ACEM-Impact Emotional Well‑being score | SWLS-C | **✓** Overall test *P* < 0.05, means properly ordered | **✓** Overall test *P* < 0.01, means properly ordered | | | **✓** Overall test *P* < 0.01, means properly ordered | Caregivers who report greater life satisfaction will have lower mean ACEM-Impact Emotional Well‑being scores than caregivers with lower satisfaction. |
|  | PROMIS Parent Proxy Depressive Symptoms SF-6a | **✓** Overall test *P* < 0.05, means properly ordered (only 1 response category had n ≥ 5) | **✓** Overall test *P* < 0.01, means properly ordered | | | **✓** Overall test *P* < 0.01, means properly ordered | Caregivers who report more depressive symptoms will have higher mean ACEM-Impact Emotional Well‑being scores than caregivers who reported fewer depressive symptoms. |
| ACEM-Impact Social Well‑being score | PGIS‑Child Impact Social | ✓ Overall tests *P* < 0.05 for Interactions with peers item and Interactions with others item  ✓ Means properly ordered for Interactions with others item only | ✓ Overall tests *P* < 0.01, means properly ordered for Interactions with peers item and Interactions with others item | | | ✓ All overall tests *P* < 0.01, means properly ordered | Caregivers who report better social interactions with peers (OS and IT visit 7), fewer limitations in what they can do with friends (OS only), or better social interactions with people other than peers (OS, IT screening and visit 7) will have lower mean ACEM-Impact Social Well‑being scores than caregivers with worse ratings. |
| ACEM-Impact Social Functioning | PGIS‑Child Impact Social | ✓ P < 0.05 for Interactions with others item and means properly ordered | ✓ All overall tests P < 0.01, means properly ordered | | | ✓ All overall tests P < 0.01, means properly ordered | Caregivers who report better social interactions with peers, fewer limitations in what they can do with friends, or better social interactions with people other than peers will have lower mean ACEM-Impact Social Functioning scores than caregivers with worse ratings. |
|  | PedsQL Social  Functioning | ✓ Overall test P < 0.01, means properly ordered | ✓ Overall test P < 0.01, means properly ordered | | | ✓ Overall test P < 0.01, means properly ordered | Caregivers with better PedsQL Social Functioning scores will have lower mean ACEM-Impact Social Functioning scores than caregivers with worse ratings. |
| ACEM-Impact Functioning score | PROMIS Parent  Proxy Upper  Extremity SF-  8a | ✓ Overall test P < 0.05, means properly ordered | ✓ Overall test P < 0.01, means properly ordered | | | ✓ Overall test P < 0.01, means properly ordered | Caregivers who report greater upper extremity interference will have higher (worse) mean ACEM-Impact Functioning scores than caregivers who reported less interference. |

ACEM–Impact = Achondroplasia Child Experience Measure-Impact;; DCGM = DISABKIDS Chronic Generic Module (DCGM-37) Proxy version; IT = interventional trial; OS = observational study; PedsQL = Pediatric Quality of Life Inventory-4.0 Generic Core Scales – Parent version; PGIS = parent global impression of severity; PROMIS = Patient-Reported Outcomes Measurement Information System-Parent Proxy; SF-10 = 10-item Short Form Health Survey for Children (proxy); SWLS-C (proxy) = Satisfaction with Life Scale – Child (proxy)[40].

Note: Mean comparisons were not conducted for groups with n < 5. SF-10 Global Health item and PGIS-Child Symptom rating subgroups were defined by the individual item response categories.

**Table S7. ACEM–Impact Test-Retest Reliability, observational and interventional trial studies**

| **Test-Retest Reliability, Baseline to Retest (Week 2), observational study** | | | |
| --- | --- | --- | --- |
| **Measure** | **Overall ICC (95% CI), n** | **No major life change ICC (95% CI), n** | **No change in PGIS ICC (95% CI), n** |
|  |  |  |  |
| **ACEM–Impact Total** | 0.76 (0.69-0.82), 177 | 0.76 (0.67-0.82), 130 | 0.75 (0.64-0.83), 85 |
| Daily Living Functioning | 0.71 (0.61-0.78), 177 | 0.75 (0.67-0.82), 130 | 0.74 (0.63-0.83), 85 |
| Physical Functioning | 0.71 (0.63-0.78), 177 | 0.75 (0.66-0.82), 130 | 0.67 (0.54-0.77), 85 |
| Emotional Well-being | 0.71 (0.62-0.78), 166 | 0.72 (0.59-0.80), 120 | 0.75 (0.63-0.83), 80 |
| Social Functioning | 0.65 (0.56-0.73), 175 | 0.62 (0.50-0.72), 128 | 0.61 (0.46-0.73), 84 |
| Social Well-being | 0.70 (0.62-0.77), 166 | 0.68 (0.57-0.76), 120 | 0.74 (0.62-0.82), 80 |
| School | 0.78 (0.69-0.85), 98 | 0.79 (0.70-0.86), 85 | 0.75 (0.59-0.85), 48 |
| **Test-Retest Reliability, interventional trial** | | | |

| **Measure** | **Screening to baseline Overall ICC (95% CI), n** | **Screening to baseline No major life change ICC (95% CI), n** | **Visit 3 to visit 4 Overall ICC (95% CI), n** | **Visit 3 to visit 4 No change in PGIS ICC (95% CI), n** |
| --- | --- | --- | --- | --- |
|  |  |  |  |  |
| **ACEM–Impact Total** | 0.79 (0.64-0.89), 38 | 0.79 (0.62-0.88), 36 | 0.83 (0.71-0.90), 45 | 0.73 (0.50-0.86), 30 |
| Daily Living Functioning | 0.69 (0.47-0.83), 37 | 0.67 (0.44-0.82), 35 | 0.80 (0.66-0.88), 45 | 0.78 (0.57-0.89), 27 |
| Physical Functioning | 0.82 (0.68-0.90), 38 | 0.82 (0.67-0.90), 36 | 0.88 (0.80-0.93), 45 | 0.76 (0.54-0.89), 26 |
| Emotional Well-being | 0.83 (0.67-0.92), 27 | 0.83 (0.66-0.92), 26 | 0.81 (0.66-0.90), 35 | 0.87 (0.72-0.94), 22 |
| Social Functioning | 0.70 (0.49-0.83), 37 | 0.68 (0.46-0.82), 36 | 0.80 (0.65-0.89), 42 | 0.80 (0.59-0.91), 24 |
| Social Well-being | 0.77 (0.59-0.88), 31 | 0.78 (0.58-0.89), 29 | 0.81 (0.65-0.89), 37 | 0.82 (0.64-0.91), 28 |
| School | 0.84 (0.61-0.94), 17 | 0.83 (0.58-0.94), 16 | 0.83 (0.63-0.93), 21 | 0.79 (0.49-0.93), 15 |

ACEM-Impact = Achondroplasia Child Experience Measure-Impact; CI = confidence interval; ICC = intraclass correlation coefficient; PGIS = parent global impression of severity.
