# Supplementary material for: Assessing signs and impacts of achondroplasia: psychometric evaluation of the Achondroplasia Child Experience Measures
Source: J Patient Rep Outcomes. 2026 Jun 19;10:162. doi: 10.1186/s41687-026-01123-z (PMC13534396; doi:10.1186/s41687-026-01123-z)
Supplement: Supplementary file 3 [file 41687_2026_1123_MOESM3_ESM.docx]

Supplementary file 3

**Assessing Signs and Impacts of Achondroplasia: Psychometric Evaluation of the Achondroplasia Child Experience Measures**

Table B. ACEM-Impact Item-Level Decisions

| **Final item stem** | **Original framework** | **Final  framework** | **Explanation** |
| --- | --- | --- | --- |
| Without assistance or an adaptive device, in the past two weeks and because of achondroplasia, how difficult was it for your child to… | | | |
| Reach objects or high places | Functioning | Daily living functioning | CFAs indicated 3 distinct clusters of Functioning items |
| Toilet themselves | Functioning | Daily living functioning |  |
| Bathe/shower, wash, and/or comb hair | Functioning | Daily living functioning |  |
| Perform tasks requiring fine motor skills (such as writing or opening jars) | Functioning | Daily living functioning |  |
| Dress or undress | Functioning | Daily living functioning |  |
| Sit for long periods of time | Functioning | Physical functioning | CFAs indicated 3 distinct clusters of Functioning items |
| Climb stairs or steps | Functioning | Physical functioning |  |
| Be physically active | Functioning | Physical functioning |  |
| Walk long distances | Functioning | Physical functioning |  |
| Run | Functioning | Physical functioning |  |
| In the past two weeks, because of achondroplasia, how often did your child… | | | |
| Have difficulty participating in class/school work or activities | Functioning | School | CFAs indicated 3 distinct clusters of Functioning items |
| Limit or modify their participation in physical education (gym class) | Functioning | School |  |
| Miss school/preschool (arrive late, leave early, or miss a full day) | Functioning | School |  |
| In the past two weeks, because of achondroplasia, how often did your child feel… | | | |
| Different from others | Emotional well‑being | Emotional well‑being |  |
| Frustrated | Emotional well‑being | Emotional well‑being |  |
| Angry | Emotional well‑being | Emotional well‑being |  |
| Sad | Emotional well‑being | Emotional well‑being |  |
| Worried | Emotional well‑being | Emotional well‑being |  |
| Embarrassed | Emotional well‑being | Emotional well‑being |  |
| In the past two weeks, because of achondroplasia, how difficult was it for your child to… | | | |
| Physically keep up with other children their age | Social well‑being | Social functioning | CFAs indicated that these 3 items clustered together, separate from the other Social Well‑being items |
| Participate in social activities (such as birthday parties, amusement parks, playdates) | Social well‑being | Social functioning |  |
| Participate in sports or physical play | Social well‑being | Social functioning |  |
| In the past two weeks, because of achondroplasia, how often did your child experience… | | | |
| Teasing/bullying | Social well‑being | Social well‑being | CFAs indicated that these 3 items clustered together, separate from the Social Functioning items |
| Negative attention in public (such as staring or pointing) | Social well‑being | Social well‑being |  |
| Being stigmatized (rejected or underestimated by others) | Social well‑being | Social well‑being |  |
| Being treated as younger than they are | Social well‑being | Not used in ACEM-Impact scoring | To be reported descriptively at the item level (Note that being treated as younger was a positive outcome for some children and a negative outcome for others) |
| In the past two weeks, because of achondroplasia, how often did your child… | | | |
| Need assistance from other people to accomplish their daily tasks (outside of school) | Need for assistance/‌adaptive devices | Not used in ACEM-Impact scoring | To be reported descriptively at the item level |
| Use adaptive devices to accomplish their daily tasks (outside of school) | Need for assistance/‌adaptive devices | Not used in ACEM-Impact scoring | To be reported descriptively at the item level |
| Need assistance from other people at school/preschool | Need for assistance/‌adaptive devices | Not used in ACEM-Impact scoring | To be reported descriptively at the item level |
| Use adaptive devices while at school/preschool | Need for assistance/‌adaptive devices | Not used in ACEM-Impact scoring | To be reported descriptively at the item level |
| Need other accommodations/adaptations at school/preschool (such as more time to take tests or get from place to place) | Need for assistance/‌adaptive devices | Not used in ACEM-Impact scoring | To be reported descriptively at the item level |

ACEM = Achondroplasia Child Experience Measures; CFA = confirmatory factor analysis.
